# Approaches to standardising the magnetic resonance image analysis of equine tendon lesions

**DOI:** 10.1002/vro2.57

**Published:** 2023-02-23

**Authors:** Carla Ulrike Doll, Melanie Bohner, Dagmar Berner, Kathrin Buettner, Carolin Horstmeier, Karsten Winter, Janina Burk

**Affiliations:** ^1^ Equine Clinic (Surgery, Orthopaedics) Justus‐Liebig‐University Giessen Giessen Germany; ^2^ Translational Centre for Regenerative Medicine (TRM) University of Leipzig Leipzig Germany; ^3^ Department for Horses Faculty of Veterinary Medicine University of Leipzig Leipzig Germany; ^4^ Equine Referral Hospital Royal Veterinary College Hawkshead Road University of London Hatfield UK; ^5^ Unit for Biomathematics and Data Processing Justus‐Liebig‐University Giessen Giessen Germany; ^6^ Institute of Anatomy University of Leipzig Leipzig Germany

## Abstract

**Background:**

Low‐field magnetic resonance imaging (MRI) has gained increasing importance to monitor equine tendon lesions. Comparing results between studies and cases is hampered, because image analysis approaches vary strongly. This study aimed to improve reliability, comparability and time efficiency of quantitative MRI image analysis.

**Methods:**

Induced tendon lesions were studied over a 24‐week period with 10 follow‐up MRI examinations. Signal intensities (SIs) of tendons, tendon lesions, cortical bone and background, as well as lesion cross‐sectional areas (CSAs) were measured. Lesion SI standardisation with different formulas was evaluated, using histological findings as reference. Different types of region of interest (ROI) for lesion SI measurement were compared. Lesion CSA measurement at different levels was evaluated, using the calculated total lesion volume as reference. Subjective lesion identification and manual CSA and SI measurements were compared to an automated, algorithm‐based approach.

**Results:**

Lesion SI standardised using a quotient of lesion and background or cortical bone SI, correlated best with histologically determined lesion severity. Lesion SI in circular ROIs correlated strongly with lesion SI in free‐hand whole‐lesion ROIs. The level of the maximum lesion CSA shifted over time; the CSA maximum correlated strongly with lesion volume. In sequences with short acquisition time, algorithm‐based automated lesion detection showed almost perfect agreement with subjective lesion identification. Automated measurement of CSA and SI was also feasible, with stronger correlation and better agreement with the manually obtained data for the SI than for the CSA.

**Conclusion:**

Our study may provide guidance for MRI image analysis of tendon healing. Reliable image analysis can be performed time‐efficiently, particularly regarding lesion SI quantification.

## INTRODUCTION

For the diagnosis of tendon lesions, ultrasonography is still standard; while the impact of magnetic resonance imaging (MRI) increases as it offers a more detailed insight in soft tissues.^1^ In recent years, MRI has become more readily available for horses in the course of the development of a low‐field MRI system that is suitable for examination of standing, sedated horses.^2^ With this system, the risks of general anaesthesia can be avoided and costs are reduced,^2^ making MRI an attractive tool for equine research and clinical diagnostic investigation.

When examining tendon lesions in low‐field MRI, T1‐weighted (T1w) and T2*w gradient recalled echo (GRE), T2w fast spin echo (FSE) and short tau inversion recovery (STIR) sequences have been used.^3^, ^4^, ^5^, ^6^, ^7^, ^8^ However, despite using the same MRI system and the same or similar sequence settings, the results are not directly comparable between studies and cases because image analysis approaches vary strongly. This pertains to the estimation of lesion signal intensity (SI) and its standardisation, as well as to the estimation of lesion dimension.

Lesion SI measurements are either performed using free‐hand drawings contouring the whole lesion,^9^, ^10^ or using different circular or rectangular region of interests (ROIs) placed within the lesion area,^2^, ^7^, ^11^ which will entail different results because signal is not homogeneous throughout the lesion. The SI standardisation is then attempted either by calculating a relative lesion SI based on quotients^4^, ^7^, ^12^ or by calculating a signal‐difference‐to‐noise ratio (SDNR)^3^; both approaches also vary as to whether the formulas use anatomical structures^3^, ^4^ and/or the background^3^, ^12^ as reference. The dimension of the lesion is either evaluated based on the cross‐sectional area (CSA) of the tendon^3^ or lesion,^3^, ^12^ or based on the tendon^3^ or lesion^3^, ^4^, ^6^ volume calculated from the CSAs determined over the whole lesion area. Generally, manual lesion contouring and measuring all images displaying the lesion should be the most comprehensive approach; however, this is time consuming and results are not completely objective because the manual drawings depend on the examiner. For this reason, automation of the measurements would be a highly attractive alternative.

The aim of this study was to improve reliability, comparability and time efficiency of quantitative MRI image analysis for monitoring of tendon healing. For this purpose, the agreement between different lesion signal and dimension quantification approaches was analysed, including an algorithm‐based automated approach.

## MATERIALS AND METHODS

### MRI images and histology samples

MRI image series and histological samples from equine superficial digital flexor tendon (SDFT) lesions were available from a previous animal study in six standardbred horses (three female and three male, 3–10 years old, 400–550 kg) in which mesenchymal stromal cell therapy had been investigated,^6^, ^13^ as approved by the local authority (Landesdirektion Leipzig, Germany; TV 34/13). The tendon lesions had been induced by combining low‐dose collagenase injection with mild mechanical tissue disruption under general anaesthesia and with subsequent pain management, as described previously.^6^ Lesions were either treated with labelled mesenchymal stem cells in serum or with serum alone. Low‐field MRI (0.27 Tesla MRI unit; Hallmarq Veterinary Imaging, Guildford, Surrey, UK) was performed at 10 time points, with the first examination before treatment and a follow‐up over 24 weeks. At each examination, the MRI sequences detailed in Table 1 were obtained. The transverse image series from forelimb tendon lesions treated with serum alone were used for image analysis in the current study. After the last examination at Week 24, the animals were euthanased and the metacarpal region of the SDFT dissected for histological examination. From the six horses, three to four histological samples per lesion (*n* = 23 in total) were assigned to the corresponding MRI images from Week 24 for the current analysis.

**TABLE 1:** FAST sequences used in low‐field magnetic resonance imaging

**FAST sequence**	**TR (ms)** **Repetition time**	**TE (ms)** **Echo time**	**Flip angle**
T1‐weighted (w) gradient recalled echo (GRE)	52	8	50°
T2*w GRE	68	13	25°
T2w fast spin echo	1544	88	90°
Short tau inversion recovery	2336	22	90°

*Note*: Each sequence was obtained with a slice thickness of 5 and 1 mm gap, with a 171 × 171 mm field of view and 256 × 256 matrix.

### Image analysis

**FIGURE 1:**
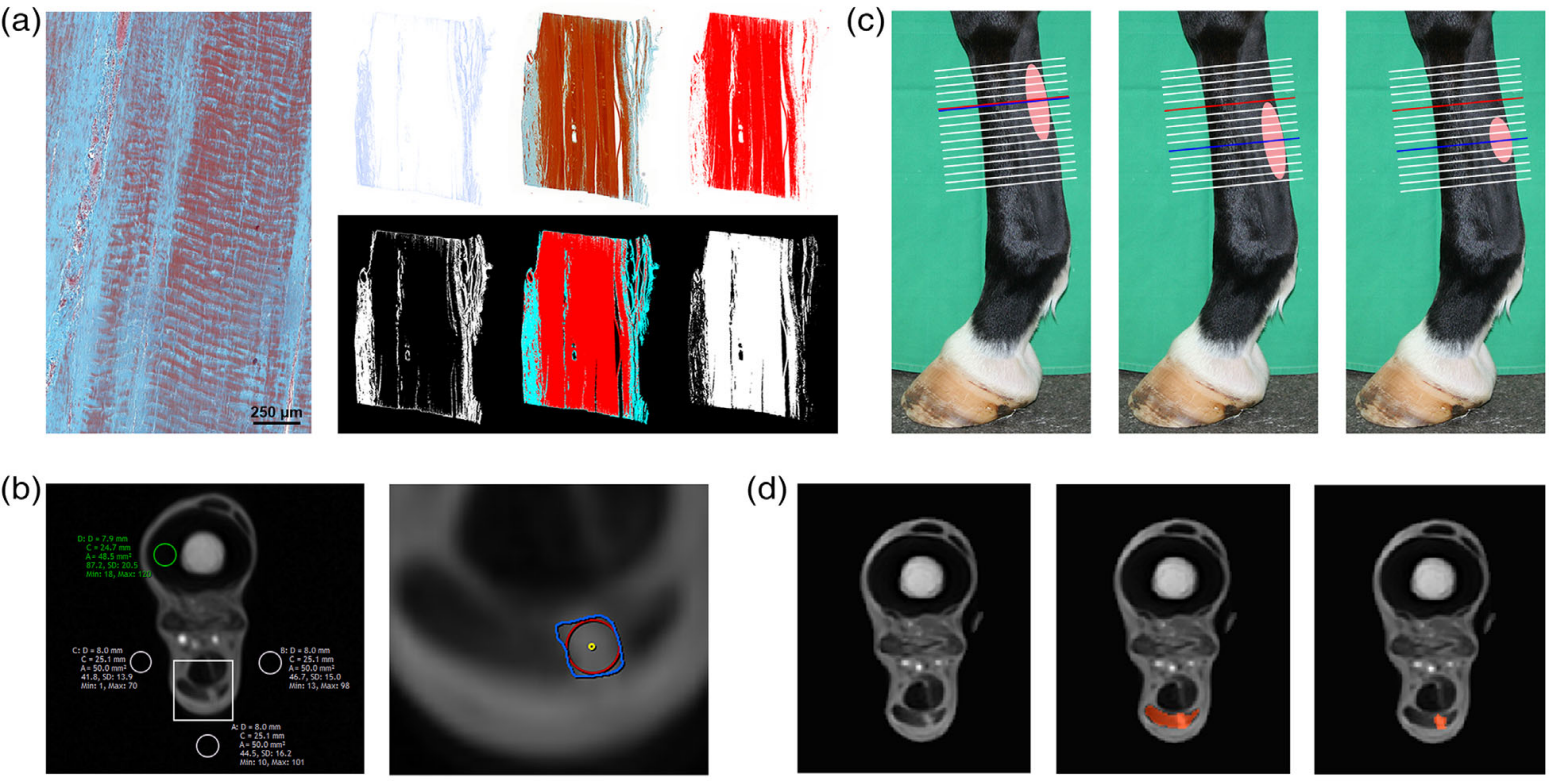
Histological evaluation and image analysis. (a) In Masson's trichrome‐stained tendon sections, the percentage of blue‐stained sample area was calculated by red and blue colour channel extraction and image segmentation. (b) In all magnetic resonance images, the signal intensity (SI) of the cortex of the metacarpal bone and the background medial, lateral and palmar to the limb were determined using 50 mm^2^ circular regions of interest (ROIs). If a lesion was present, its SI and cross‐sectional area (CSA) were measured by free‐hand drawing (blue). The lesion SI was also obtained using the largest possible circular ROI fitting into the lesion (red) and a 1 mm^2^ circular ROI in the centre of the lesion (yellow). (c) The lesion CSA was determined at the level of the maximum injury at the first examination for all examination time points (CSA fixed; red), as well as at the level of maximum injury at each examination time point (CSA maximum; blue). (d) Automated image analysis was performed using Mathematica software. Within the manually drawn superficial digital flexor tendon ROI, the lesion was determined automatically by an algorithm.

For histology, Masson's trichrome‐stained longitudinal sections were digitalised in a slide scanner (Pannoramic SCAN, 3DHISTECH, Budapest, Hungary). Using Mathematica software (Version 10.3.0.0; Wolfram Research Inc., Mathematica, Champaign, IL, USA), the red and blue colour channels were extracted from the acquired images and the respectively stained regions were segmented^14^ to calculate the percentage of blue pixels within each image as a measure for tendon lesion severity (Figure 1a).^15^

Measurements on MRI images were first performed manually in Synedra View Personal 16 (Version 16.0.0.2; Synedra Information Technologies GmbH, Innsbruck, Austria). The following measurements were repeated three times in all images and mean values for each image were used for further analysis. To allow for SI standardisation, the SI of the cortex of the metacarpal bone was determined based on a circular 50 mm^2^ ROI, and the SI of the background was determined using three circular 50 mm^2^ ROIs positioned lateral, medial and palmar to the limb. The SI of the SDFT was measured based on free‐hand drawing. The lesion SI was obtained based on three different ROIs: the whole‐lesion ROI as defined by free‐hand drawing, the largest possible circular ROI fitting into the lesion and a 1 mm^2^ circular ROI in the centre of the lesion (Figure 1b). The CSA of the lesion was determined by free‐hand drawing and the maximum lesion levels were determined for each time point (Figure 1c).

Additionally, MRI image analysis was automated using Mathematica software. At first, an ROI defining the SDFT was manually drawn in each MRI image, using an image processing program (GIMP 2.8, GNU Image Manipulation Program, GIMPTeam, Verden, Germany). In Mathematica, each image was then segmented using a locally adaptive algorithm with a filter width of 30 pixels to determine the lesion within the SDFT ROI. Lesion CSA and SI were then obtained using a binary mask (Figure 1d).

### Data analysis

First, different approaches to background correction to standardise the SI between images were evaluated. The whole lesion SI and cortical bone or background SI data obtained in Synedra were substituted in each of the following four equations:

(1) $$\begin{array}{ccc} & & \text{Signal-Difference-to-Noise-Ratio}\;\left(\mathrm{SDNR}\right)\\ & & \;\;=\;\frac{\mathrm{SI}\left(\mathrm{lesion}\right)-\mathrm{SI}\left(\mathrm{SDFT}\right)}{\mathrm{SD}\left(\mathrm{background}\right)} \end{array}$$

(2) $$\mathrm{SDNR}=\frac{\mathrm{SI}\left(\mathrm{lesion}\right)-\mathrm{SI}\left(\mathrm{SDFT}\right)}{\mathrm{SD}\left(\mathrm{cortical}\;\mathrm{bone}\right)}$$

(3) $$\;\mathrm{Relative}\;\mathrm{SI}=\frac{\mathrm{SI}\left(\mathrm{lesion}\right)}{\mathrm{SI}\left(\mathrm{background}\right)}$$

(4) $$\mathrm{Relative}\;\mathrm{SI}=\frac{\mathrm{SI}\left(\mathrm{lesion}\right)}{\mathrm{SI}\left(\mathrm{corticalis}\right)}$$

The approach leading to the standardised SI that corresponded best to lesion severity in the respective histological sections was to be used in the subsequent analyses. This part of the analysis was performed for the images in the T1w GRE sequence obtained on the last MRI examination at Week 24, for which corresponding histology was available. For each of the 23 histological sections, the mean SI from the respective corresponding MR images was used for statistical analysis.

Next, it was analysed to what extent the measurement of lesion SI using circular ROIs, either the largest possible circular ROI or the 1 mm^2^ circular ROI, corresponded to the whole‐lesion SI determined by free‐hand drawing (Figure 1b). The SI was standardised using Equation (3), and the images from all MRI examinations and sequences that were of sufficient quality were included (T1w GRE: *n* = 640 images; T2w FSE: *n* = 230 images; T2* GRE: *n* = 521 images; STIR: *n* = 73 images).

Furthermore, it was evaluated whether the lesion CSA from one image per series was representative for the volume of the total lesion. For this purpose, two different approaches for choosing the level to determine the lesion CSA were investigated. On the one hand, the maximum lesion level at the first MRI examination was identified and the same level was used to assess lesion CSA at all further time points (CSA fixed). On the other hand, the maximum lesion level was identified for each MRI examination time point consecutively (CSA maximum) (Figure 1c). Besides analysing the respective resulting CSA versus total lesion volume, the shift in maximum lesion level to proximal or distal over time was evaluated by calculating the distances between the CSA maximum and CSA fixed levels for each horse and time point. This analysis was done using T1w GRE sequence image series from all MRI examinations (*n* = 60 image series from the lesion areas).

Finally, to assess whether image analysis could be automated, the agreement between subjective lesion detection during the manual measurement procedures and automated lesion detection based on the algorithm programmed with Mathematica software was compared (Figure 1d). This was done using the images from all MRI examinations and sequences (T1w GRE: *n* = 650 images; T2w FSE: *n* = 400 images; T2* GRE: *n* = 590 images; STIR: *n* = 400 images). Then, for all of these images that displayed a lesion and were of sufficient image quality, the manual and automated whole‐lesion CSA and SI measurements were compared (T1w GRE: *n* = 643 images; T2w FSE: *n* = 286 images; T2* GRE: *n* = 568 images; STIR: *n* = 244 images).

### Statistical analysis

For the statistical analysis, SPSS Statistics 28 (IBM, Ehningen, Germany) was used. Spearman's rank correlations were calculated for the four SI standardisation formulas versus the percentage of blue pixels in Masson's trichrome staining; for the SI determined by the two circular ROIs versus the SI measured by free‐hand whole lesion drawing; for the CSA fixed and CSA maximum versus the lesion volume; and for the manual versus the automated measurements of lesion CSA and SI. The backtransformed logarithmised limits of agreement, reflecting the ratio of data obtained by two measurement methods,^16^, ^17^ were additionally calculated for the comparison of the SI measurements using the two circular ROIs to the SI measured by free‐hand whole‐lesion drawing; also for the comparison of the manual to the automated measurements of the lesion CSA and SI. For the latter, as a qualitative analysis, the agreement between manual (subjective) and automated lesion detection was calculated as well. The level of significance was set at *p* < 0.05.

## RESULTS

### Standardisation of the lesion signal intensity

The standardisation of the lesion SI, in the T1w GRE sequence, using Equations (3) and (4) for relative SI yielded results, correlated better with the histological lesion severity than when using the Equations (1) and (2) for SDNR. The correlation between relative SI and percentage of blue pixels in histology was significant with *r* = 0.608 and *p* = 0.002 (Equation 3) and *r* = 0.513 and *p* = 0.012 (Equation 4). Equation (3) was used for subsequent analyses.

### Representative circular regions of interest for lesion signal intensity measurements

**FIGURE 2:**
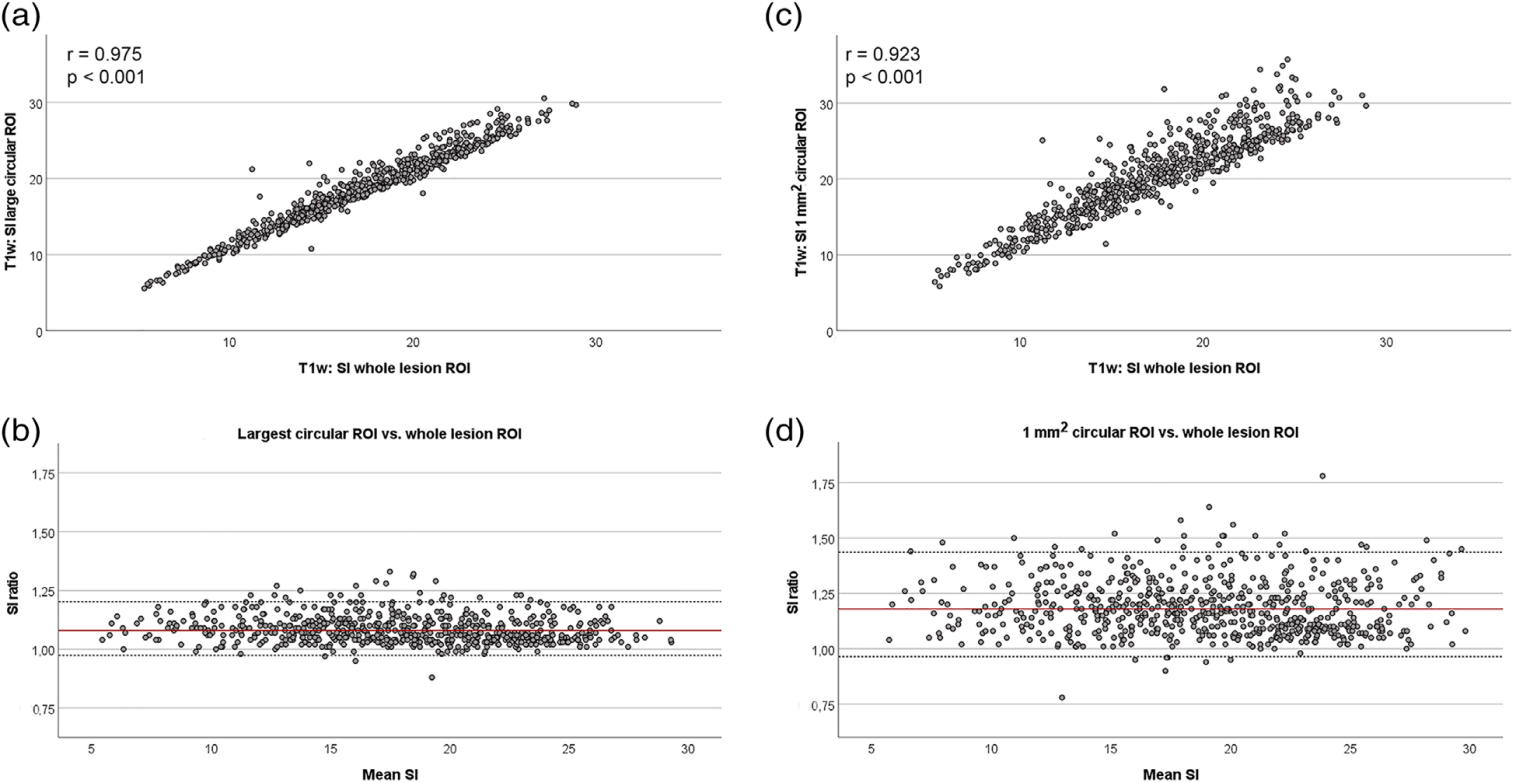
Lesion signal intensity (SI) measured in different types of regions of interest (ROIs). Lesion SI was measured either in the largest possible circular ROI, in the centrally placed 1 mm^2^ ROI or the free‐hand drawn whole‐lesion ROI. The SI measured in both circular ROIs correlated strongly with the SI in the hand‐drawn whole‐lesion ROI. Correlation (a and c; Spearman's rank correlation) and agreement (b and d; Bland‐Altman analysis with backtransformed logarithmised data) with the free‐hand drawn ROI were higher for the large circular ROI than for the 1 mm^2^ circular ROI. Data were obtained from *n* = 640 T1‐weighted gradient recalled echo images. Data from other sequences are shown in Supporting Information S1.

The SI measurements using circular ROIs correlated strongly with the SI measurements using the hand‐drawn whole‐lesion ROIs. This applied to both types of circular ROIs. However, the dot plots demonstrated a more linear relationship between the SI of the large circular ROI and the SI of the whole‐lesion ROI (Figure 2a,c; Figure S1). Furthermore, the ratio between the latter was closer to 1 (Figure 2b,d; Figure S1) underlining that the agreement was better when using the largest possible circular ROI as compared to the 1 mm^2^ circular ROI. This was constant throughout the different MRI sequences analysed (Figure 2; Figure S1).

### Representative level for lesion cross‐sectional area measurements

**FIGURE 3:**
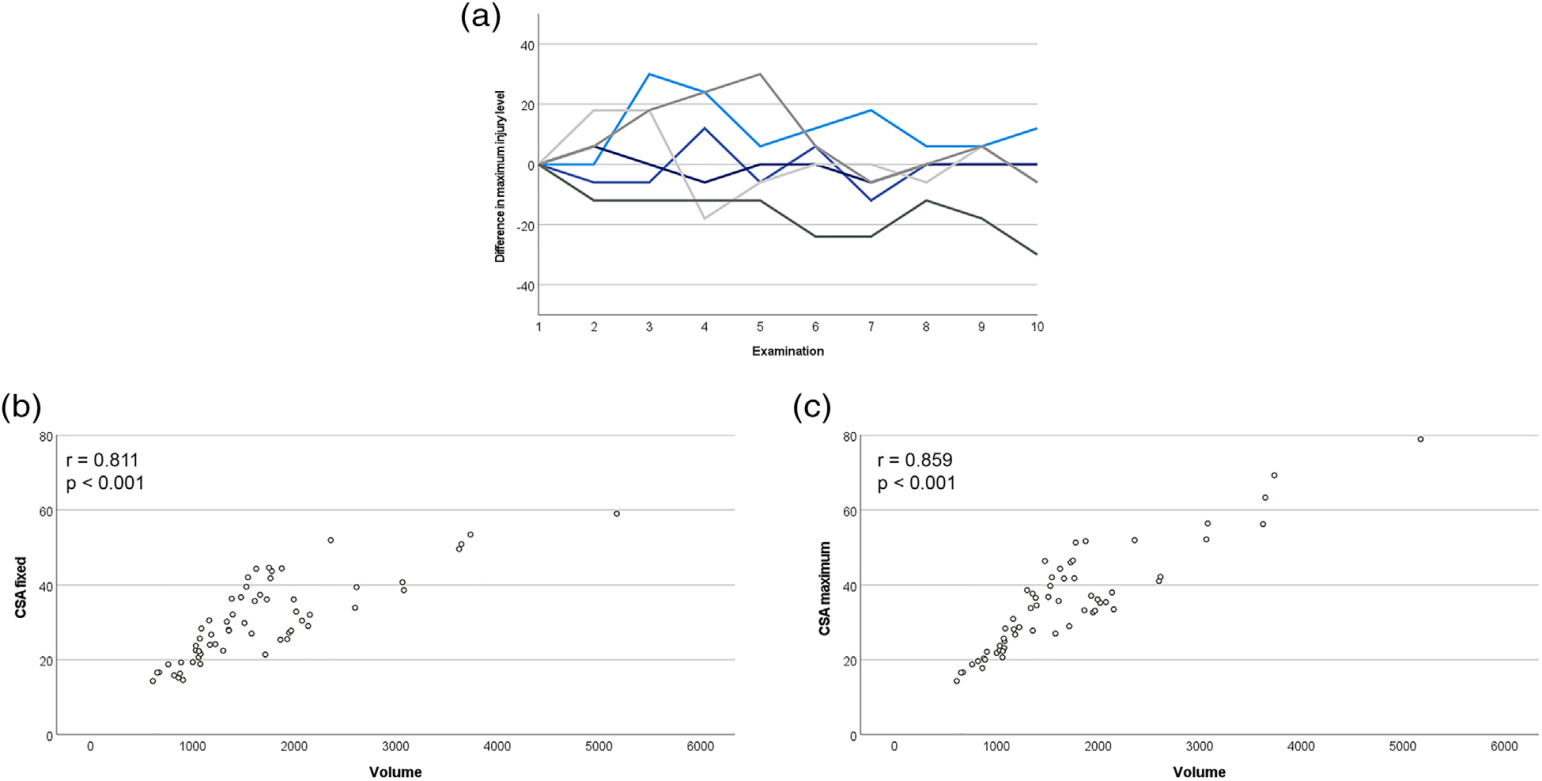
Lesion cross‐sectional area (CSA) measured at different levels and lesion volume. (a) The maximum lesion level shifted over time as compared to the first examination. The line plot shows the difference in maximum injury level in mm at each examination over 24 weeks (2–10) as compared to the first examination (1) for each individual horse. Positive values represent a shift to proximal, negative values represent a shift to distal. (b and c) The dot plots show the lesion CSA of the position that had the maximum CSA at the first examination (CSA fixed, shown in b) and the maximum CSA at each successive examination (CSA maximum, shown in c), versus lesion volume. The correlation with the volume of the tendon lesion was stronger for the CSA maximum than for the CSA fixed. Lesion volume is given in mm^3^, CSA in mm^2^. Data were obtained from *n* = 60 T1‐weighted gradient recalled echo image series.

The maximum lesion level did not remain constant during follow‐up; there was no general trend that it shifted to either proximal or distal (Figure 3a). The CSA fixed as well as the CSA maximum correlated strongly with the lesion volume in the T1w GRE sequence. Yet, the relationship was more linear for CSA maximum than for CSA fixed (Figure 3b,c).

### Automated lesion detection and measurement

**FIGURE 4:**
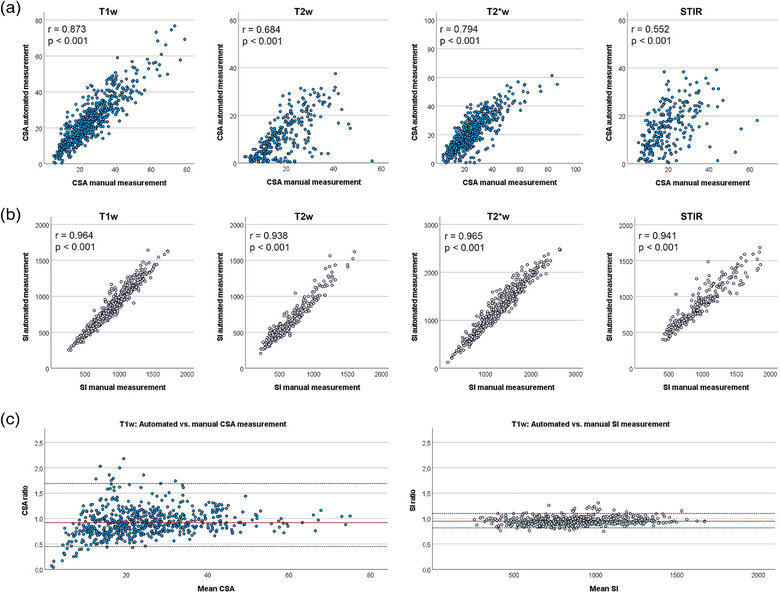
Automated compared to manual lesion measurements. (a) Correlation of the manual and automated lesion cross‐sectional area (CSA) measurements was high in the T1‐weighted gradient recalled echo (T1w GRE) and T2*w GRE sequences, while only moderate in T2w fast spin echo and short tau inversion recovery (STIR) sequences. (b) The correlation of signal intensity (SI) measurements was very strong in every sequence. (c) The ratio of automatically to manually measured CSAs and SIs in T1w GRE sequence, reflected by backtransformed logarithmised data in the Bland–Altman plot, revealed slightly lower values in the automated measurements and a better agreement for SI than for CSA. Bland–Altman plots for other sequences are shown in Supporting Information S2. Data were obtained from *n* = 643 T1w GRE images, *n* = 286 T2w FSE images, *n* = 568 T2* GRE images and *n* = 244 STIR images.

The automated detection and measurement of tendon lesions were highly reliable in sequences with high signal‐to‐noise ratio, but less reliable in those with poorer image quality. The agreement between manual and automated lesion detection was almost perfect for the T1w GRE (98.92%) and the T2*w GRE sequences (97.63%), whereas only substantial for the T2w FSE (74.50%) and STIR sequences (75.00%). Correspondingly, the CSA manual and automated measurements correlated more strongly in the T1w GRE and T2*w GRE sequences, whereas the correlation was only substantial in the T2w FSE and moderate in the STIR sequence (Figure 4a). The manual and automated SI measurements showed a very strong correlation in all sequences (Figure 4b). The Bland–Altman analysis of the backtransformed logarithmised data further revealed that the lesion CSA measured in the automated manner was overall slightly lower than when measured manually. It also confirmed the better agreement for the SI measurements than for the CSA measurements. This was similar in all sequences (Figure 4c; Figure S2).

## DISCUSSION

The aim of the study was to optimise MRI examinations of tendon lesions with regard to the image analysis. First, the reliability of the standardisation of the SI was taken into account; second, approaches for reducing time and efforts with regard to the ROI drawing and the area to be scanned and measured during follow‐up examinations were evaluated. Finally, we investigated whether the manual measurement of the CSA and determination of the SI of the lesion could be replaced by an automated measurement.

Histology was used as reference for the first analyses, which focused on standardising the SI. Although corresponding MRI images and histological sections were obtained from the exact same tendon regions, the MRI scans were transverse while the histology sections were longitudinal. These different orientations, which are most representative for MRI and histology, respectively, were deliberately chosen to yield most information from the respective diagnostic modality. Nevertheless, it needs to be acknowledged that direct overlays from MRI and histology images could not be obtained and analysed.

For standardising the SI, the current analysis suggested that simple quotients reflected the severity of the tendon lesions better than SDNR calculations. However, it did not play a major role whether the background outside the limb or the cortical bone were used as reference in the formulas. The most important aspect regarding the latter is the biological invariability of the reference. This was suggested to apply to the cortex of the metacarpus,^4^ although the SI of the background might be more suitable due to possible bone remodelling, especially for long‐term studies over several months. Past studies have standardised the SI in different ways. The SDNR was obtained by calculating the difference between the SI within the lesion of the SDFT and the SI of the healthy deep digital flexor tendon and dividing it by the SD of the background,^3^ similar as performed in Equation (1). In most other studies, the SI of SDFT lesions was standardised using quotients according to the current Equation (3), using the background as reference,^7^, ^12^ or according to the current Equation (4), using the cortical bone as reference.^4^ Based on the present data, calculating a quotient is the preferable approach, which in addition requires fewer measurements per image.

For measuring the raw SI, the current data indicated that whole‐lesion ROIs could be replaced by circular ROIs. In most previous studies, free‐hand whole‐lesion ROIs were used,^3^, ^4^, ^7^ which is reasonable when these ROIs are used to measure lesion CSA at the same time. However, using standardised ROIs is less time consuming for SI measurement if whole‐lesion ROIs are not drawn for other reasons.^18^ Furthermore, this approach depends less on the person performing the measurements. Here, we compared the largest circular ROI fitting into the respective lesion and a 1 mm^2^ circular ROI placed in the centre of the lesion with the free‐hand whole‐lesion ROI for SI measurements. The largest possible circular ROI showed the best agreement with the whole‐lesion ROI, which is plausible because the SI of tendon lesions is usually highest in the centre and decreases in the lesion periphery, which is only reflected by large ROIs. However, although the SI in very small ROIs could be influenced more by possible artefacts within the image, the SI measurements using the 1 mm^2^ ROI also correlated strongly with the whole‐lesion SI measurements in all sequences. Therefore, this approach can be considered as reliable as well, while using a small circular ROI allows the performance of follow‐up measurements with a fixed‐size ROI, while lesion dimension decreases over time, which again saves time and might be more objective. Nevertheless, these results are obviously not interchangeable and directly comparable with those obtained using large ROIs.

To estimate the development of the lesion dimension over time, our data suggested that maximum lesion CSA measurements were representative for the whole lesion dimension. In previous studies, either lesion volume^3^, ^4^, ^6^ or the maximum lesion CSA^7^, ^12^ were used for follow‐up. However, it could be assumed that any CSA does not necessarily reflect the proximo‐distal dimension of the lesion. Here, the maximum CSA, as identified for each examination time point, the CSA of the position that had displayed the maximum CSA at the first examination, referred to as CSA fixed, were compared to the volume of the entire lesion. The purpose of this attempt was to determine whether follow‐up scans and image analyses should always include the entire lesion region or whether they could be limited to the level that displayed the lesion maximum at the time of the first examination. The latter would be more time‐efficient, including a shorter sedation time during the MRI examination. Both positions for CSA measurements showed a strong correlation with the lesion volume. However, the CSA maximum, identified for each examination, appeared to be more representative for the lesion volume than the fixed CSA. This was due to the position of the maximum of the lesion changing over time, at which we observed shifting of the maximum lesion level to more distal regions in some horses, while also shifting to more proximal regions in other horses. Therefore, it cannot be recommended to focus only on the initial maximum lesion level during the follow‐up, because it is not possible to predict the development of the lesion in its entirety.

Finally, an attempt was made to replace the manual measurements with a programmed algorithm. In principle, it is conceivable that a program that reacts to brightness levels of image pixels is able to evaluate MRI images and, in particular, recognises tendon lesions. In human Achilles tendons, the volume of the tendon has already been calculated by masking the tendon using a seed growing technique and calculating the tendon volume by an algorithm based on the voxel volume.^19^ Furthermore, we have previously used the current automated approach to characterise equine tendon lesion progression.^6^ In the current study, the reliability of this approach was validated for the identification of tendon lesions as well as for measuring their CSA and SI. Depending on the sequence, there was a substantial to almost perfect agreement between the subjective and the automated lesion detection. Occasional mismatch was mainly due to motion artefacts occurring in sequences with a longer acquisition time (T2w FSE and STIR). Regarding the CSA measurements, the algorithm designated a smaller area as the lesion than when manually drawn in all sequences. This was marginal in the T1w GRE and T2*w GRE sequences, while differences and variation within data were more evident in the T2w FSE and STIR sequences. Therefore, although images with significant artefacts had been excluded from the analyses, image quality was still an issue for automated CSA measurements. However, automated SI measurements showed a very good agreement and correlation with the manually obtained data in all sequences. Based on the current data, we can recommend algorithm‐based automated tendon lesion quantification, although only when image quality is adequate for reliable lesion identification.

The current study provides a basis for recommendations for MRI image analysis of tendon healing. Both time efficiency and reliability of image analyses could be improved with regard to SI quantification, by calculating the standardised SI as a simple quotient with the background SI as denominator; by replacing hand‐drawn whole‐lesion ROIs with circular ROIs. However, a reduction of the region to be scanned during follow‐up based on the initial maximum lesion level cannot be recommended. Finally, automated image analysis showed promising results, although it relies on adequate image quality. If high‐quality images are available, their algorithm‐based analysis can be strongly recommended because it is time efficient and highly objective.

## AUTHOR CONTRIBUTIONS

Dagmar Berner and Janina Burk conceived and designed the project. Carolin Horstmeier acquired the MRI image series and prepared the histological sections. Melanie Bohner, Carla Ulrike Doll and Karsten Winter analysed the images. Kathrin Buettner and Carla Ulrike Doll analysed the data. Janina Burk and Carla Ulrike Doll drafted the manuscript. All authors have critically revised and approved the final version of the manuscript.

## CONFLICTS OF INTEREST

The authors declare they have no conflicts of interest.

## ETHICS STATEMENT

The authors confirm that the ethical policies of the journal, as noted on the journal's author guidelines page, have been adhered to. All procedures of the underlying animal study had been approved by the responsible authority (Landesdirektion Leipzig, TV 34/13).

## Supporting information

Supporting Information

## Data Availability

All data relevant to the study are included in the article. The datasets used and analysed during the current study are available from the corresponding author upon reasonable request.
