# Supplementary material for: Approaches to standardising the magnetic resonance image analysis of equine tendon lesions
Source: Vet Rec Open. 2023 Feb 23;10(1):e257. doi: 10.1002/vro2.57 (PMC9950356; doi:10.1002/vro2.57)
Supplement: Supplementary file 1 — Supporting Information [file VRO2-10-e257-s001.docx]

# **Supporting Information**

## Supporting Information 1 (S1)

## Signal intensity (SI) measured different types of regions of interest (ROIs)

Lesion signal intensity (SI) was measured in free-hand drawn regions of interest (ROIs), as well as in the largest possible circular ROIs fitting into the lesion, and in 1 mm² circular ROIs placed in the centre of the lesion, to elucidate if the circular ROIs would be representative for the whole lesion.

The SI measured in both circular ROIs correlated strongly with the SI in the hand-drawn whole lesion ROI. Correlation (A and C; Spearman’s rank correlation) and agreement (B and D; Bland-Altman analysis with backtransformed logarithmised data) with the free-hand drawn ROI were higher for the large circular ROI than for the 1 mm^2^ circular ROI.

Data from T1-weighted (w) gradient recalled echo images are displayed in the main article.


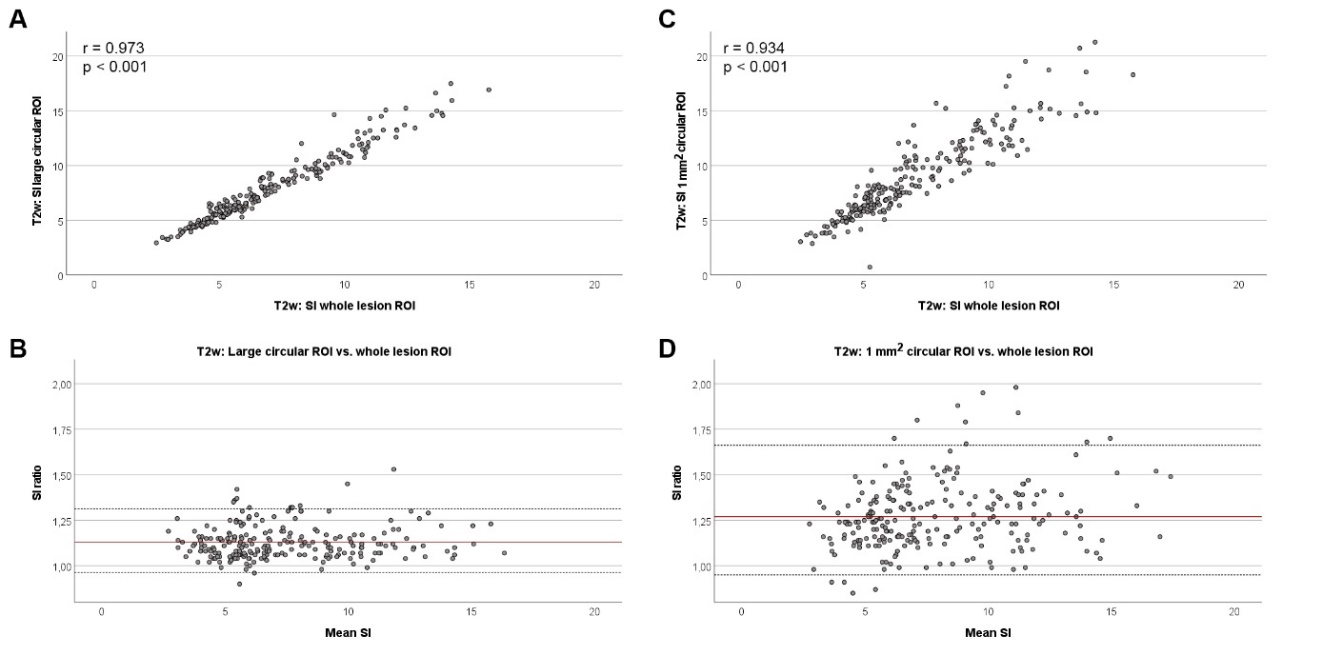


## Figure S1.1 Data from n = 230 T2-weighted (w) fast spin echo images


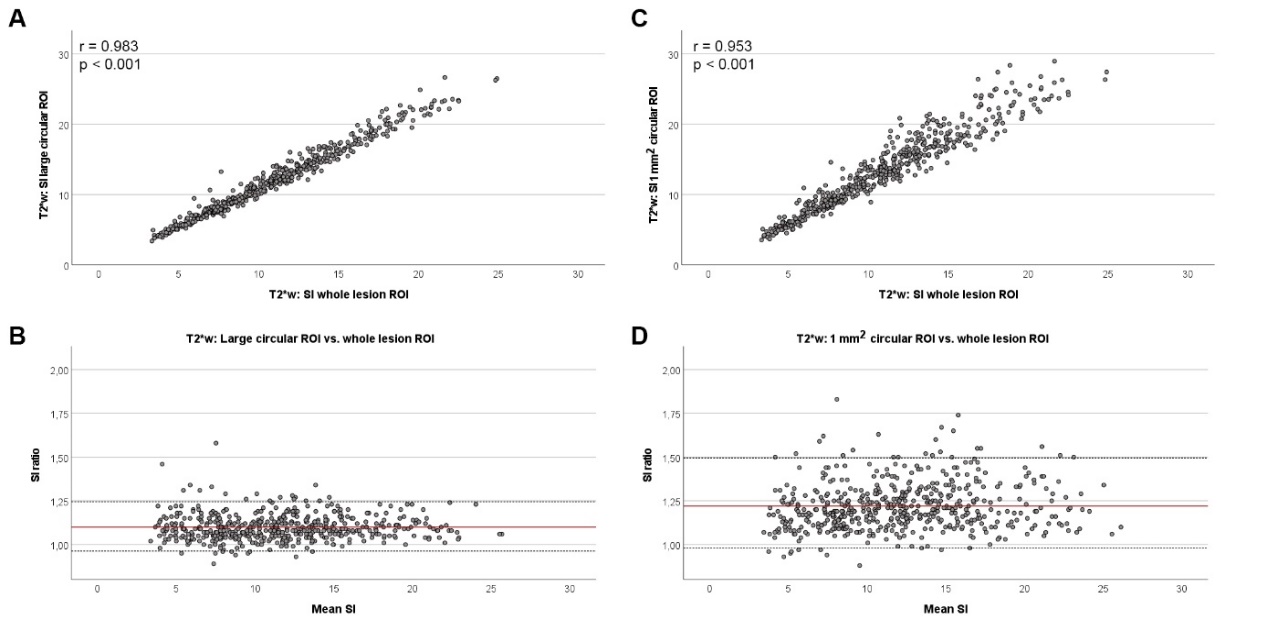


## Figure S1.2 Data from n = 521 T2*-weighted (w) gradient recalled echo images


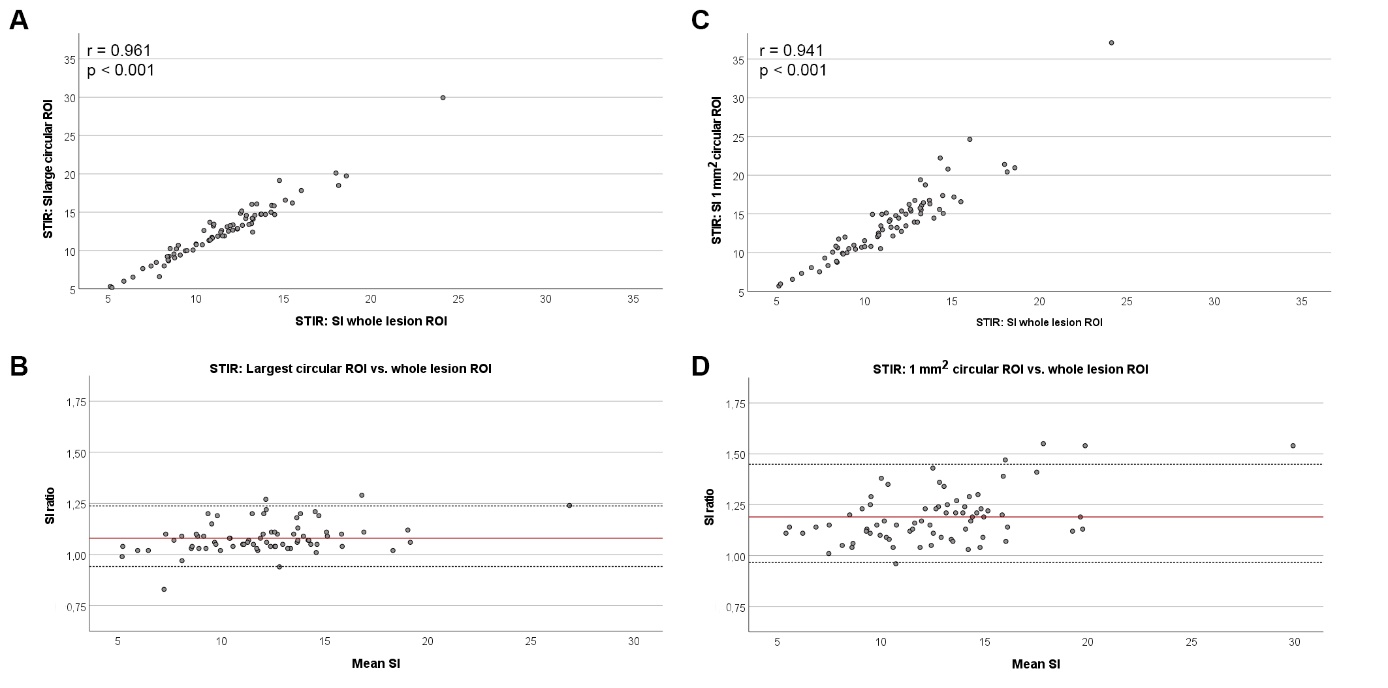


## Figure S1.3 Data from n = 73 short tau inversion recovery (STIR) images

## Supporting Information 2 (S2)

## Agreement between manual and automated lesion cross-sectional areas (CSA) and Signal Intensity (SI) measurements

Lesion cross-sectional area (CSA) and signal intensity (SI) were determined either with manual measurements using Synedra software, or by an algorithm-based measurement using Mathematica software, to evaluate whether MRI image analysis could be automated.


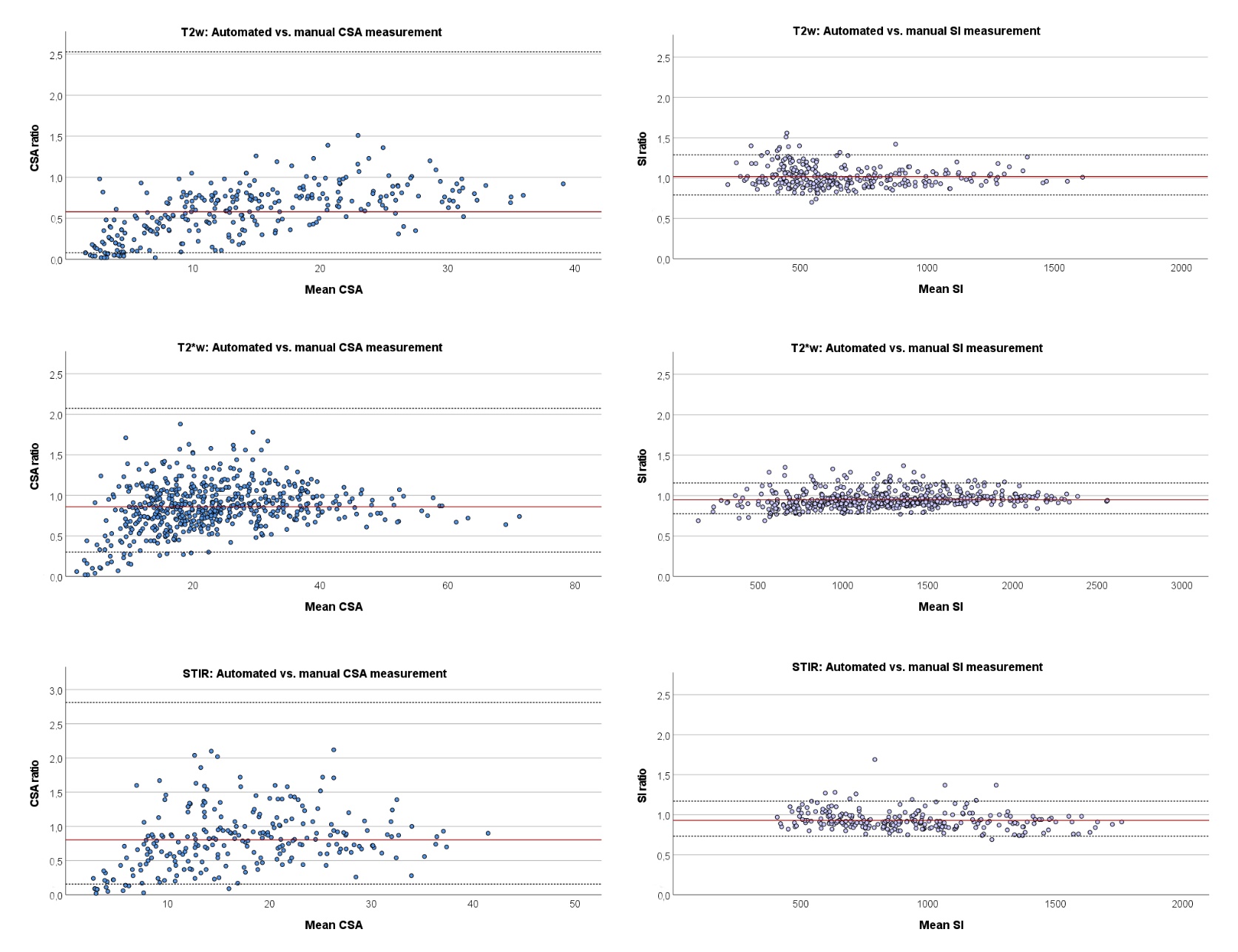


The ratio of automatically to manually measured CSAs and SIs, reflected by backtransformed logarithmised data in the Bland-Altman plot, revealed slightly lower values in the automated measurements and a better agreement for SI than for CSA.

Bland-Altman plots for T1-weighted gradient recalled echo images are displayed in the main article.

Data were obtained from n = 286 T2-weighted fast spin echo (T2w) images, n = 568 T2*-weighted gradient recalled echo (T2*w) images and n = 244 short tau inversion recovery (STIR) images.
